# Psychometric properties of the Iranian version of mindful eating questionnaire in women who seeking weight reduction

**DOI:** 10.1186/s40337-018-0220-4

**Published:** 2018-11-02

**Authors:** Zahra Abbaspoor, Nahid Javadifar, Mahsa Miryan, Parvin Abedi

**Affiliations:** 1 Midwifery Department, Reproductive Health Promotion Research Center, Ahvaz Jundishapur University of Medical Sciences, Ahvaz, Iran; 2 Reproductive Health Promotion Research Center, Ahvaz Jundishapur University of Medical Sciences, Ahvaz, Iran; 3 student of nutrition, Faculty of paramedicine, Ahvaz Jundishapur University of Medical sciences, Ahvaz, Iran; 4 Menopause, Andropause Research Center, Ahvaz Jundishapur University of Medical Sciences, Ahvaz, Iran

**Keywords:** Mindfulness eating, Questionnaires, Reliability, Validity

## Abstract

**Background:**

The aim of the present study was to test the validity, reliability and factor structure of the original Mindful Eating Questionnaire (MEQ) for use in an Iranian population.

**Methods:**

This was a cross-sectional study conducted on 150 women who attended four athletic gyms and met the inclusion criteria in Ahvaz city in July of 2015. After linguistic validation of the Iranian version of the MEQ, the content validity ratio (CVR) and content validity index (CVI) were assessed by an expert panel. Then, exploratory factor analysis (EFA) was performed on the scale constructs and scale reliability (internal consistency and test-retest reliability) was assessed with respect to the psychometric properties of the scale.

**Results:**

The CVR and CVI scores for the MEQ were 0.89 and 0.93, respectively. EFA loaded all 28-items with a 5-factor solution (‘awareness’, ‘distraction’, ‘disinhibition’, ‘emotional response’ and ‘external cues’) that jointly accounted for 53.78% of the observed variance. The results of the EFA supported the item ‘When a restaurant portion is too large, I stop eating when I’m full’ being placed in the external cues rather than the disinhibition subscale. This displacement improved the reliability coefficient for this subscale.

The results of internal consistency analysis, including Cronbach’s alpha (ranging from 0.73 to 0.81) and intraclass correlation coefficients (ranging from 0.73 to 0.91) were satisfactory.

**Conclusions:**

The Persian version of the MEQ appears to be valid and reliable; therefore, it can be an effective tool in designing mindfulness-based interventions for the treatment of individuals with eating disorders, overweight and obesity in an Iranian population.

## Plain English summary

The prevalence of obesity has increased worldwide over the past decade and according to the World Health Organization, more than 650 million (13%) individuals over the age 18 years are obese. The prevalence of obesity also has increased in Iran in the recent years and reached to 21.3% in population with age > 18 years. Some disordered eating behaviours may be important factors in the prevalence of obesity.

Mindfulness is described as a non-judgmental awareness of the present moment and is effective in decreasing psychological symptoms including depression and anxiety in individuals with obesity. Several scales specifically measure mindfulness and are widely used in applied settings, but there are limited specific scales to measure mindfulness with respect to eating behaviors. The mindful eating questionnaire (MEQ) is the first scale measures mindful eating. Because of a high prevalence of overweight and obesity in Iran, this study aimed to evaluate the Mindful Eating Questionnaire (MEQ) for use in an Iranian population.

## Background

The prevalence of obesity has increased worldwide over the past decade and according to the World Health Organization, more than 650 million (13%) individuals over the age 18 years are obese [1]. The prevalence of obesity also has increased in Iran in the recent years and reached to 21.3% in population with age > 18 years [2]. Some disordered eating behaviors, such as binge eating (uncontrolled eating of a large amount of food in short periods of time) may be important factors in the prevalence of obesity [3, 4]. Psychological distress plays a role in this type of eating behavior [5]. Mindfulness approaches, such as non-judgmental awareness of the present moment, are described extensively in the scientific literature as effective interventions in decreasing psychological symptoms, including depression and anxiety [6] and also in reducing binge-type eating in individuals with obesity [7]. Several scales specifically measure mindfulness [8–11] and are widely used in applied settings, but there are limited specific scales to measure mindfulness with respect to eating behaviours. The mindful eating questionnaire (MEQ) as the first scale with good reliability and validity, measures mindful eating [12].

The MEQ is a 28 item questionnaire with five domains namely; awareness (7 questions), distraction (3 questions), disinhibition (8 questions), emotional response (4 questions) and external cue (4 questions). The total possible scores for subscales are in range of 7–28, 3–12, 8–32, 4–16 and 4–16, respectively (13).

Pintado-Cucarella et al., in their study on 216 participants used MEQ and assessed the relationship of mindful eating and body mass index. Their results showed that low score of mindful eating was significantly contributed to overweight, anxiety and binge eating [14].

Taylor et al., in their study used MEQ questionnaire and found that self-compassion is significantly related to mindful eating and body mass index [15] Also Clementi et al., assessed the abbreviated form of MEQ (including 20 questions) on 1067 samples in Italy and found that this version is a valid and reliable means to use by clinicians and researchers [16]. Framson et al., in their study found that the lower score of MEQ was significantly related to body mass index> 30 kg/m^2^ in women (13).

The prevalence of obesity in Iran is relatively high (21.3%) [2], and investigation into healthful dietary behavior and related health outcomes in this population is important.

To the best of our knowledge, no study on mindfulness and eating topic have been conducted in Iran, therefore this study aimed to evaluate the psychometric properties of the Iranian version of Mindful Eating Questionnaire. We believe that this research will contribute to the existing knowledge on the topic and will provide an instrument for use by nutritionists in clinical settings as well as by researchers to evaluate the effects of mindful eating skills on healthier eating behavior in Iran.

## Methods

### The questionnaires

The MEQ is a 28-item questionnaire that contains five subscales: awareness (7 items), distraction (3 items), disinhibition (8 items), emotional response (4 items) and external cues (6 items). Response categories are rated on a four-point Likert scale, where a score of 1 suggests an eating behavior that is performed never/rarely and 4 indicates a behavior that is performed usually/always. The scores on questions 1, 2, 6, 7, 9, 11, 13, 17, 18, 19, 27 and 28 should be reversed. The total possible scores for awareness (7 items), distraction (3 items), disinhibition (8 items), emotional response (4 items) and external cues (4 items) subscales are in the ranges of 7–28, 3–12, 8–32, 4–16 and 4–16, respectively [13].

### Translation and cultural adaptation

A forward-backward procedure was applied to translate the English version of the MEQ into the Persian language. Initially, two forward and conceptual translations were produced by two independent translators who were not aware that the tool would be subsequently translated back into English. The researchers then compared the two translations and produced the first draft of the Persian version of the questionnaire. Two other translators, who were unaware of the questionnaire, back translated the Persian questionnaire into the English language. Subsequently, in the synthesis step, the research team evaluated the final English version against the original version and, together with a specialist in psychometrics, reviewed the entire translation processes. Consequently, a test of face validity was performed to provide a pre-final version of the questionnaire. Finally, an agreement in terms of semantic, idiomatic, conceptual and cultural equivalence was reached and the final version of the questionnaire was provided. In the new questionnaire compared with original version, there were only minor modifications to the wording and additional descriptions in parentheses to improve understanding by the target group.

### Design and data collection

#### Participants and procedure

This was a cross-sectional study conducted on 150 women who attended four gyms to perform exercise in Ahvaz city during July 2015. This group was selected because behavioral eating disorders, obesity and higher intake of food are more common in women especially in those who seeking weight loss [17, 18].

In the gyms, at the end of an exercise program and after the study aims were explained and written consent to participate was obtained, women were asked to complete the MEQ questionnaire.

Women were eligible if they met each of the following criteria: participation in any recreational exercise in the gym, functional literacy and a willingness to commit to this research study. Exclusion criteria were: professional athletes, being on diets under the supervision of a nutritionist, using any kind of pharmaceutical weight control, having any severe mood disorder controlled by pharmaceuticals and current known substance abuse. Research questionnaires were completed by participants at a separate location in the gym following the conclusion of exercise.

### Statistical analysis

#### Face validity

The establishment of qualitative and quantitative face validity can improve the assistance of respondents in completing a questionnaire, identify any ambiguities in the wording of items and identify any inappropriate items [19–21]. To establish the qualitative face validity and to determine how long the questionnaire takes to complete, the MEQ was completed through interview with 10 women to ensure the linguistic and conceptual equivalence of the translations. On the basis of the results of the pilot study and research team opinions, necessary changes were made, the MEQ was modified as appropriate and the final questionnaire was obtained. In addition to the determination of item importance, a quantitative face validity test was conducted to measure the impact scores of items using a formula.

#### Content validity

To calculate the qualitative content validity, 10 experts in the fields of nutrition, psychology and reproductive health who were familiar with the psychometric process were asked to provide their views on the accuracy of item content in written form. They also checked item position, grammar and the use of appropriate words in phrases.

Furthermore, in quantitative content validity collected from a panel of 10 experts, item importance and accuracy using a three-point rating scale was examined using the content validity ratio (CVR) and evaluating the design of the items, including relevance, clarity and simplicity, by content validity index (CVI). CVR can measure between − 1.0 and 1.0. The closer to 1.0 the CVR is, the more essential the object is considered to be. In this study the content evaluation panel was composed of 10 experts and a minimum CVR of .62 is required based on Lawshe table. Only those items with CVR meeting this minimum are retained in the questionnaire [22]. The CVI was calculated according to the Lawshe table on the basis of the ratings by the experts who rated each item [23]. Judgment on each item is made as follows: if I-CVI is less than 70%, the item is not acceptable and will be omitted, the I-CVI between 70 and 79% is questionable and the item needs to revise and the I-CVI higher than 79% is appropriate [24, 25].

#### Construct validity

##### Factor analysis

**Fig. 1:**
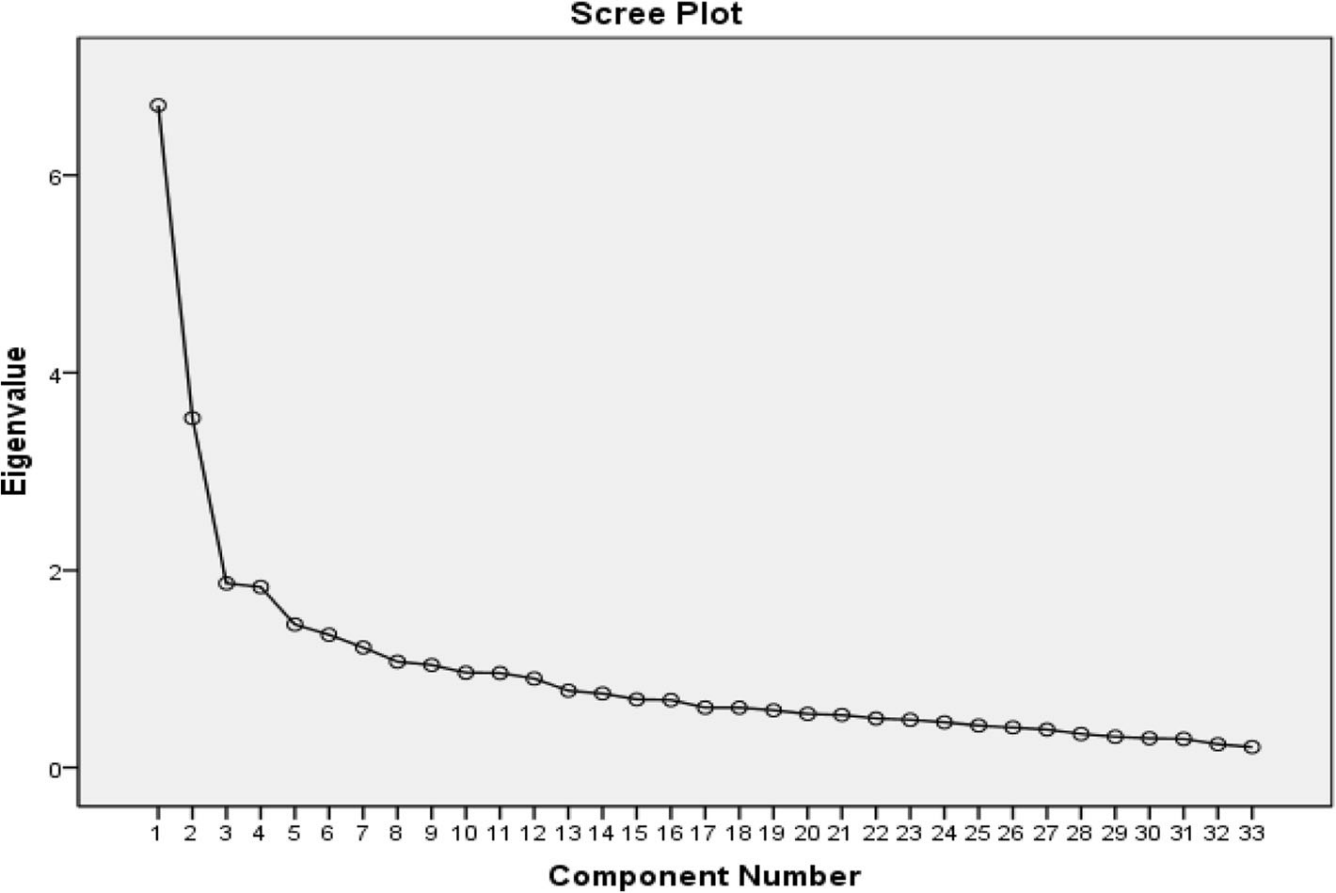
Scree plot of eigenvalues for principal components analysis of MEQ

Factor analysis is a statistical method that enables the underlying subscales of a questionnaire to be determined [20]. Exploratory factor analysis have recommended in order to establish equivalence. This technique has been widely used specially in validation of the factor structure of the frequently translated questionnaires in a different sample or to perform adaptation of a questionnaire to another language [26]. In the present study the factor structure of the MEQ was determined by exploratory factor analysis (EFA), utilizing principal component analysis with varimax rotation [27]. Varimax is the most popular rotation method and usually produces simpler solution and easier to interpret. Varimax maximizes the sum of the variances of the squared loadings correlation between variables and factors [28]. To determine the best structure, an eigenvalue greater than 1.2 and a factor loading equal to or greater than 0.4 and scree plot were applied [29, 30] (Fig. 1).

#### Concurrent validity

The focus of the current available instruments used to screen eating disorders is on behaviors and diagnostic criteria but some of them such as Eating disorders belief questionnaire (EDBQ) and Three Factor Eating Questionnaire (TFEQ) are based on the drive from thinness and the fear of fatness [31]. The Eating Disorder Belief Questionnaire (EDBQ) is a relatively brief questionnaire intended for use within the eating disorder population. It is a multi-dimensional and self-report measure with 32 items and four subscales as follows: [1] negative self-beliefs, [3] weight and shape as a means to acceptance by others, [4] weight and shape as a means to self-acceptance and [5] control over eating. The negative self-beliefs subscale appears to measure generic beliefs associated with depression. The other three subscales appear to measure beliefs specific to eating disorders [32]. The EDBQ’s ability to distinguish assumptions about weight and shape from assumptions about eating is important, as it has been proposed that the core psychopathology of eating disorders lies in the personal meaning attached to weight and shape [33]. This questionnaire has been validated in the Persian language [34].

#### Reliability

The reliability of a questionnaire is measured as the variance in a score that reflects the true score, rather than random error; that is, the extent to which measures give consistent or accurate results. Reliability has two common forms: internal consistency or homogeneity and test-retest reliability methods. Test-retest and internal consistency were used to assess the reliability of the MEQ. The internal consistency was assessed using the coefficient Cronbach’s alpha, which ranges from 0 to 1, and values equal to or >0.70 for a scale indicate a satisfactory internal consistency [35]. Test-retest reliability measures stability over time, by administering the same test to the same subjects at two points in time. To evaluate the test-retest reliability, a total of 30 participants, randomly selected using convenience sampling, in the same manner as the initial subject recruitment, completed the Persian MEQ again four weeks later. The test-retest reliability of the scale was estimated using the intraclass correlation coefficient (ICC). The following category was selected for the interpretation of agreement levels: 00–0.2 as small, 0.21–0.40 as fair, 0.41–0.60 as moderate, 0.61–0.80 as substantial and 0.81–1 as almost perfect [36]. Statistical analysis was performed using Statistical Package for the Social Sciences 21.0 (SPSS, Inc., Chicago, IL, USA). *P* values < 0.05 were considered to indicate significant results.

## Results

### Participants

In all, 150 women (5 times the number of items) were included in the study [37, 38]. The mean age of the women was 27.99 (SD ± 10.83) (13–59) years. The correlation between BMI and MEQ total score was not significant, but there was an inverse significant correlation between BMI and awareness dimension of MEQ questionnaire (*p* = 0.01).

Socio-demographic and clinical characteristic of the women are presented in Table 1.

**Table 1:** Characteristics of the study population, *n* = 150

Age (years), mean ± SD	27.99 ± 10.83
BMI (kg/m2) mean ± SD	24.57 ± 6.05
Education, *n* (%)	
Primary school or less	3 (2)
Secondary school	4 (2.7)
Diploma	31 (20.7)
University	95 (63.3)
BMI (kg/m2)), *n* %)	
< 19	6 (4)
19–24.9	76 (50.7)
25–30	35 (23.3)
>30	24 (16)
OCP users, *n* (%)	
Yes	19 (12.7)
No	123 (82)
Menopause, *n* (%)	
Yes	7 (4.7)
No	135 (90)

### Face validity

Almost all participants indicated that the questionnaire was easy to read and understand, but minor changes were suggested to improve upon clarity. The completion time was less than 5 min. The results of the impact factor test showed that all items had an item impact score ≥ 1.5, identifying them as important in the target group.

### Content validity

The CVR in this study for the total scale was 0.89 and for all items was greater than 0.62 according to 10 experts, based on the Lawshe Table [23], indicating the necessity and importance of the presence of relevant items in the scale. In measuring CVI, the scores of 26 items (92.8%) were ≥ 0.79 and for the total scale, 0.93, indicating a satisfactory content validity. On the basis of expert suggestions, some items were modified slightly.

The scores of two items, “if it doesn’t cost much more, I get the larger sized food or drink regardless of how hungry I feel” and “I notice when I’m eating from a dish of candy just because it’s there” were between 0.70 and 0.78. The CVI was evaluated once more using 10 experts. The I-CVI between 70 and 79% is questionable and the item needs to be revised (24).

### Factor analysis

The MEQ was analysed by principal component factor analysis with varimax rotation. The overall Kaiser-Meyer-Olkin measure of sampling adequacy was 0.79. The Bartlett’s test for sphericity produced a significant result (*p* < 0.001), indicating that the variables were correlated with one another. Hence, our preliminary analyses confirmed the appropriateness of principal component factor analysis for the data. The MEQ was found to have five factors. The percentage variances explained by rotated factor matrices ranged from to 8–12% per factor, with five factors explaining 53.78% of the overall variance. Percentages refer to the variance explained by each factor as follows: awareness 12.77%, disinhibition 11.75%, emotional 10.58%, external 9.81% and distraction 8.86%. Factor loading after rotation of each item is shown in Table 2.

**Table 2:** Factor loadings from the MEQ principal component analysis

^a^Item	Factor				
	1	2	3	4	5
**A**^b^21. Before I eat I take a moment to appreciate	**.829**	.110	.103	.054	.017
**A** 20. I notice when foods and drinks are too sweet	.**783**	.084	.180	.065	.040
**A** 22. I taste every bite of food that I eat.	**.762**	.085	.047	.088	.203
**A **16. I appreciate the way my food looks on my plate.	**.733**	.266	.018	.076	.009
**A** 12. When eating a pleasant meal, I notice if …	**.596**	.315	.117	.020	.246
**A** 26. I notice when the food I eat affects my emotional…	**.582**	.015	.335	.025	.138
**A** 10. I notice when there are subtle flavors	**.467**	.136	.048	.087	.104
**DI** 11. If there are leftovers that I like, I take	.101	**.694**	.116	.238	.255
**DI** 2. When I eat at “all you can eat” buffets	.048	**.654**	.335	.092	.083
**DI** 15. I stop eating when I’m full even	.121	**.653**	.078	.135	.279
**DI** 7. When I’m eating one of my favorite .	.052	**.647**	.161	.136	.016
**DI** 9. If it doesn’t cost much more, I get…	.056	**.542**	.123	.216	.301
**DI** 18. If there’s good food at a party, I’ll continue…	.038	**.506**	.447	.265	.161
**DI** 25. When I’m at a restaurant, I can tell…	.314	**.502**	.063	.042	.041
**ER** 27. I have trouble not eating ice cream…	.098	.016	**.777**	.071	.184
**ER** 13. I snack without noticing that I am eating.	.044	.058	**.640**	.308	.370
**ER**17. When I’m feeling stressed at work,	.107	.291	**.634**	.309	.144
**ER** 19. When I’m sad, I eat to feel better.	.135	.234	**.617**	.306	.185
**EC** 24. I notice when I’m eating from a dish	.036	.319	**.453**	.246	.189
**EC** 5. When a restaurant portion is too large,	.048	.011	**.406**	.144	.291
**EC** 8. I notice when just going into a movie.	.058	.150	.033	**.746**	.041
**EC**14. When I eat a big meal, I notice…	.069	.074	.203	**.717**	.194
**EC** 4. I recognize when food advertisements make…	.004	.201	.118	**.684**	.084
**EC** 23. I recognize when I’m eating and not hungry.	.211	.102	.159	**.607**	.159
**EC** 3. At a party where there is a lot of good food,	.053	.258	.223	**.461**	**.461**
**DT** 6. My thoughts tend to wander while I am eating	.039	.229	.120	.066	**.804**
**DT** 28. I think about things I need to do while I am eating.	.101	.140	.029	.049	**.741**
**DT** 1. I eat so quickly that I don’t taste what I’m eating.	.014	.242	.316	.018	**.664**

^a^Items: *A* Awareness, *DI* Disinhibition, *ER* Emotional Response, *EC* External Cues, *DT* Distraction

^b^Question numbers

### Concurrent validity

The concurrent validity of the MEQ was calculated using Pearson’s product-moment correlations with EDBQ as another relevant measure of acceptance. The results of correlational analysis between MEQ and EDBQ subscales indicated a significant correlation (*p* ≤ 0.001) (Table 3). All of the MEQ subscales correlated negatively with all of the EDBQ factors, except for awareness and external cues. The mindfulness eating scale that showed the highest correlations with EDBQ measures was the emotional response factor.

**Table 3:** Correlations between mindfulness eating and eating disorder belief factors

Awareness disinhibition emotional response external cues distraction					
EDBQ					
Negative self-beliefs	.024	−.140	−.211^b^	.141	−.161
Weight acceptance by others	.015	−.310^a^	−.319^a^	.205^b^	−.246^b^
Weight to self-acceptance	.138	−.377^a^	−.344^a^	.248^b^	−.229^b^
Control over eating	.032	−.298^a^	−.348^a^	.182	−.290^a^
Awareness		.030	−.301^a^	−.178^b^	.216^a^
Disinhibition			.518^a^	−.536^a^	.399^a^
Emotional response				−.523^a^	.513^a^
External cues					−.250^a^

^a^Correlation is significant at the 0.01 level (2-tailed)

^b^Correlation is significant at the 0.05 level (2-tailed)

### Reliability

Cronbach’s alpha ranged from 0.73 to 0.81 and for the scale as a whole was 0.66, indicating a reasonable reliability. The ICC for the MEQ subscales was satisfactory (ICC ranged from 0.73 to 0.91; *P* < 0.05) for each subscale, the data are presented in Table 4. Among the mindfulness eating subscales, correlational analyses showed significant positive relationships, unless the correlation between the external cues and disinhibition and emotional response subscales (*p* ≤ 0.001) and the correlation between the distraction and awareness (*p* < 0.008) and external cues subscales (*p* < 0.002). There was no significant correlation between the disinhibition and awareness subscales. The strongest positive relationships were between the factors emotional response and disinhibiting (*p* < 0.001) (Table 3).

**Table 4:** Descriptive statistics, Cronbach’s alpha and ICC of the MEQ subscales

Subscales	Cronbach’s alpha			
	Alpha of subscales in English version	Alpha of subscales with one item Exchanged^a^	Mean ± SD	ICC (95% confidence interval)
Awareness	(0.74)7 items	0.81	7 3.10 ± 0.32	0.73*
Disinhibition	(0.83)8 items	0.751 (7 items)	2.64 ± 0.54	0.77*
Emotional response	(0.71)4 items	0.818	3.27 ± 0.32	0.82*
External cues	(0.70)6 items	0.732 (7 items)	3.57 ± 0.56	0.91*
Distraction	(0.64)3 items	0.769	2.39 ± 0.45	0.88*

^a^When a restaurant portion is too large, I stop eating when I’m full

*P-value *< 0.05*

## Discussion

The purpose of this study was to analyze the psychometric properties of the MEQ in an Iranian sample. This paper reports the translation procedure, structure, validity and reliability of the MEQ in Iran. The transfer of instruments that are conceptually and functionally appropriate into another language is a complex process that requires broad research [39]; therefore, in the psychometric process, we followed the guidelines for cross-cultural adjustment of psychometric measurement and obtained cultural and conceptual equivalence. Thus, like the original version, the Iranian translation of the MEQ was culturally applicable to the Iranian people.

For measures of content validity, we used both quantitative and qualitative methods to assess face and content validity in order to take advantage of this combination in evaluating construct validity [40]. According to estimates of face validity, there were no substantial changes made to the original version and only minor changes in wording or additional descriptions of some items were made to improve upon the participants’ understanding. The CVI score for most items was ≥0.80 and for two items with inappropriate scores, the items were measured again after revision and corrected. CVR results revealed the necessity and importance of the presence of all items. In addition,

EFA results supported the factorial structure with the five constructs reported by Framson et al. (2009) [16]. The discrepancy was ‘when a restaurant portion is too large, I stop eating when I’m full’, which was originally assigned by the developers to the disinhibition factor, but was loaded on the external cues factor with an improved alpha reliability coefficient in the present study. The results reflected that the MEQ had adequate reliability indicators and internal consistency of all subscales was high (alpha from 0.73 to 0.81), similar to the values of alpha reported by Framson et al. (2009) (range, 0.64–0.83) [16]. The reliability of the MEQ summary scale was 0.66, similar to that reported by Framson (0.64). In general, a satisfactory level of internal consistency is considered a Cronbach’s α ≥ 0.7 [41]. The test-retest reliability of the MEQ with a four-week interval was found to be high. Using the intraclass correlation coefficients test, all subscales achieved a high correlation (≥ 0.7), indicating that in a stable health state over time, the MEQ produces constant results from participants. In studies by Framson et al. (2009) and Gebolla et al. (2012), the test-retest reliability was not measured [16, 42]. In correlations with other scales, the MEQ correlated negatively with all of the EDBQ factors, except awareness and external cues. As mentioned by Framson et al., the awareness domain was taken from the affective sensitivity domain, which involves awareness of internal states, and external cues subscale. In the original study, there was a combination of some items loaded in this domain [16].

### Limitations of the study

Since this study was conducted on women who attending gyms and participated in a physical activity program, this limits the generalizability of the results to all women and also to men. Further since athletic women have more mindful eating and these women mostly have higher education, we could not use these results for women with lower education that may have more eating and weight disorders. In the present study we benefited from EDBQ to test the concurrent validity of MEQ but it is recommended that further research conduct to include other measures of mindfulness such as Mindful Attention Awareness Scale (MAAS) to provide stronger support for concurrent validity [43].

## Conclusions

Psychometric properties of the Iranian version of the MEQ with five-factor structure were approved through qualitative and quantitative face and content validity, reliability and acceptability for the target group. Using the Persian version of MEQ should be done with caution, because it was only tested among women with physical activity. So first we suggest subsequent studies in both genders of Iranian population and also in women without physical activity. Using this questionnaire will be useful in for improvements of eating behaviors and stress reduction as well as nutrition research and it can also be used in mindfulness-based interventions for dietary behavioral disorders and obesity.

## Abbreviations

CVI: Content validity index
CVR: Content validity ratio
EDBQ: Eating disorder belief questionnaire
EFA: Exploratory factor analysis
ICC: Intraclass correlation coefficient
MEQ: Mindful eating questionnaire
